# Establishing Cardiopulmonary Bypass Before General Anesthesia in a Patient Undergoing Right Atrial Myxoma Resection

**DOI:** 10.7759/cureus.105976

**Published:** 2026-03-27

**Authors:** Kamilla Beisenova, Andrew Vogel, Schafer Paladichuk, Russell Carter, Tyler J Wallen, Matthew Mullen

**Affiliations:** 1 Department of Anatomy, University of New England College of Osteopathic Medicine, Biddeford, USA; 2 Department of Cardiothoracic Surgery, University of North Carolina at Chapel Hill, Chapel Hill, USA; 3 Department of Anatomy, Pacific Northwest University of Health Sciences, Yakima, USA; 4 Department of Cardiothoracic Surgery, Geisinger Health System, Wilkes-Barre, USA

**Keywords:** cardiopulmonary bypass circuit, general anesthesia induction, hemodynamic stability, large atrial myxoma, tricuspid valve obstruction

## Abstract

We describe a case of establishing cardiopulmonary bypass (CPB) before general anesthesia in a person who has an obstructive right atrial myxoma protruding into the right ventricle (RV). A 27-year-old female endorsed shortness of breath on exertion, dry cough, and palpitations, with a cardiac MRI exhibiting a severely enlarged right atrium (RA) with a 65 mm x 40 mm mass. The mass obstructed the tricuspid valve from closing and extended into the RV, leading to an ejection fraction (EF) of 49%. The obstruction caused concern for cardiovascular collapse due to hypotension and bradycardia that may be provoked by inducing general anesthesia. Under local sedation, the femoral vessels were cannulated, CPB was initiated, and the patient received general anesthesia. A median sternotomy was completed, and traditional CPB was established. After the heart was arrested, the RA was opened, and the mass was resected along with a full-thickness section of the RA. This case report supports preoperative CPB for patients undergoing general anesthesia to correct a cardiac mass that is obstructing central venous return.

## Introduction

Cardiac myxomas are the most common primary cardiac tumors and are typically managed with surgical resection under conventional induction of general anesthesia followed by cardiopulmonary bypass (CPB). However, large right atrial myxomas that prolapse across the tricuspid valve may significantly obstruct venous return and ventricular filling, placing patients at risk of acute hemodynamic collapse during induction due to venodilation and reduced preload. Previous reports have focused primarily on intraoperative anesthetic management strategies to maintain stability, including cautious fluid administration and vasoactive support [1]. Isolated cases in other high-risk pathologies, such as mediastinal masses or ruptured aortic aneurysms (excluding atrial myxomas), have described initiation of CPB prior to induction [2]. The present case is unique in demonstrating a planned, pre-induction femoral CPB strategy specifically to mitigate anticipated cardiovascular instability from a tricuspid-obstructing right atrial myxoma, thereby expanding peri-operative management options for similar high-risk presentations.

## Case presentation

**Figure 1:**
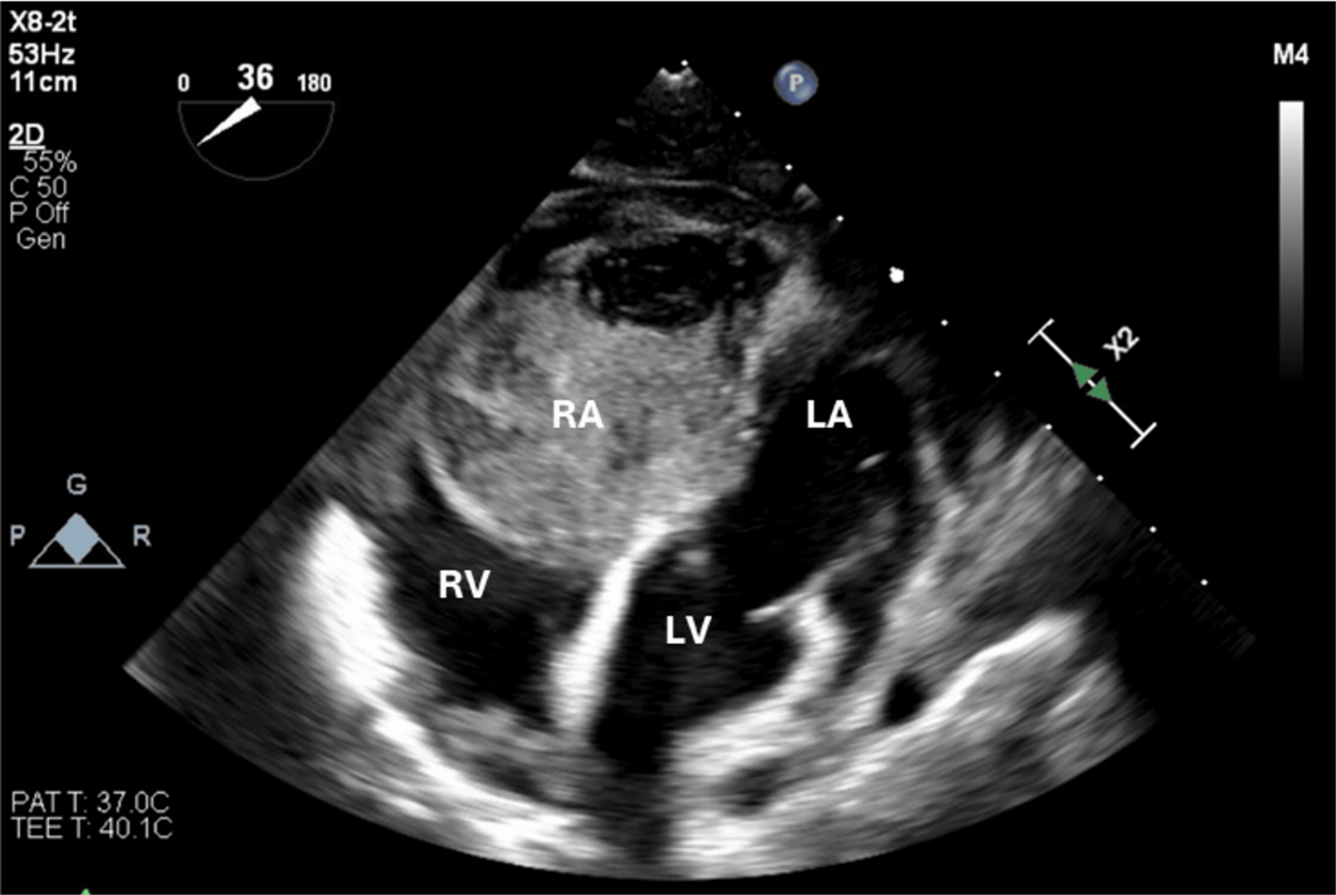
Transesophageal echocardiogram depicting the right atrial myxoma. RA: right atrium; RV: right ventricle; LA: left atrium; LV: left ventricle

A 27-year-old female presented to the hospital following symptomatic tachycardia, prominent neck veins, and an abnormal cardiac imaging result obtained during an outpatient evaluation (Figure 1).

**Figure 2:**
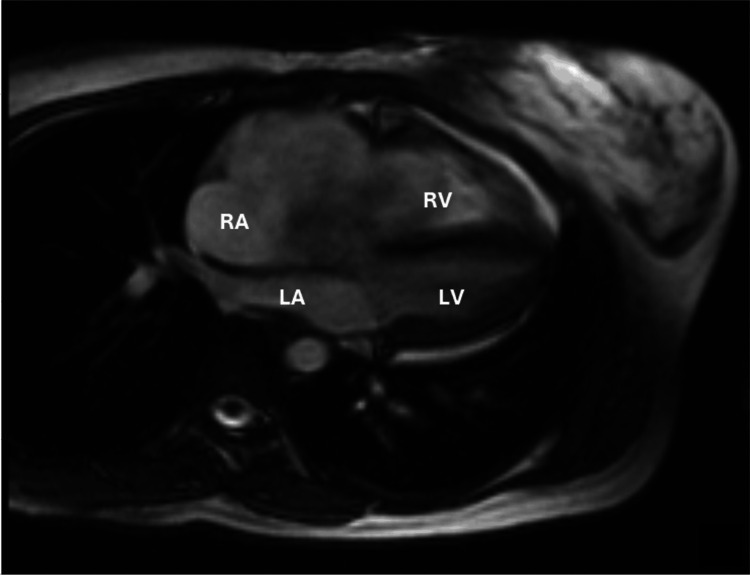
Sagittal cardiac magnetic resonance imaging demonstrating a right atrial myxoma compressing the right ventricle. RA: right atrium; RV: right ventricle; LA: left atrium; LV: left ventricle

She reported several months of palpitations, dry cough, and exertional shortness of breath. The patient had a history of tuberculosis, which had been treated. On first encounter, the patient had already presented with the diagnosis of an atrial myxoma. However, it is important to note that in addition to an atrial myxoma, other differential diagnoses include carcinoid heart disease, collagen vascular disease, pulmonary embolism, and tricuspid regurgitation/stenosis. A cardiac magnetic resonance imaging (MRI) revealed a severely enlarged right atrium (RA) containing a 65 mm x 40 mm mass (Figure 2).

**Figure 3:**
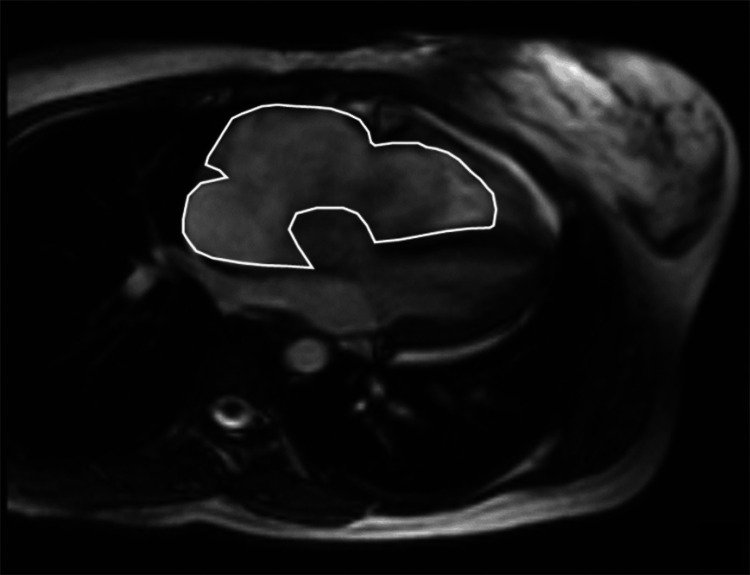
Sagittal cardiac magnetic resonance imaging demonstrating a right atrial myxoma, highlighted with a white border to delineate the mass.

The mass was obstructing the tricuspid valve, preventing proper closure, and extended into the right ventricle (RV) (Figure 3).

**Figure 4:**
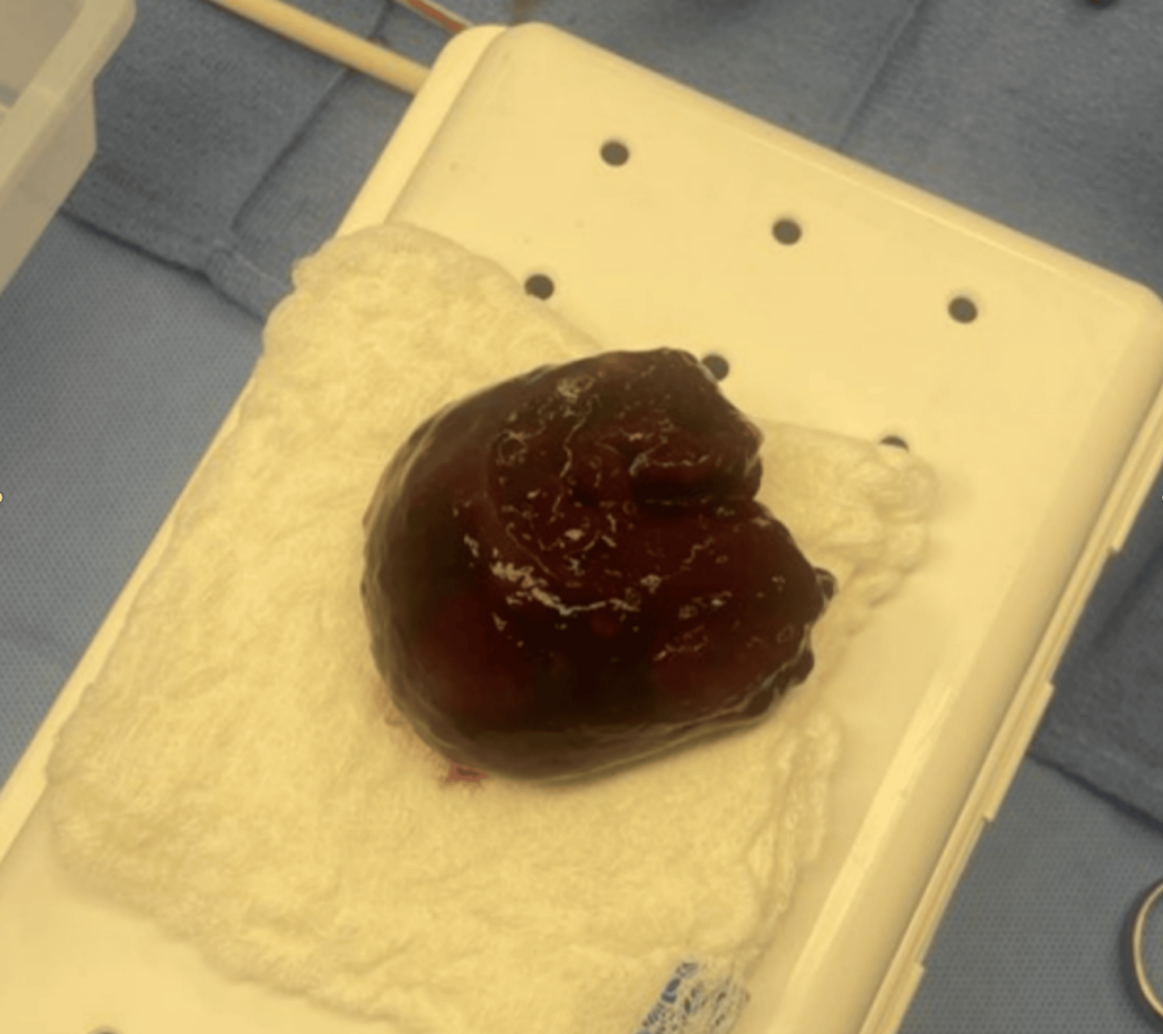
Excised right atrial myxoma.

The obstruction resulted in a mildly reduced ejection fraction (EF) of 49%. The reduced EF raised significant concern for potential cardiovascular collapse due to the venodilatory effect of general anesthesia induction with subsequent reduction in preload. Given this risk, the clinical team decided to initiate CPB before administering general anesthesia. The patient was placed under light sedation, and a right groin incision was made to establish femoral access for CPB. A cannula was placed in the common femoral artery for the outflow circuit, while another was inserted into the common femoral vein and advanced into the RA for venous drainage. Once CPB was successfully initiated, general anesthesia was administered. Subsequently, a median sternotomy was performed, and the patient was transitioned to central CPB with cannulation of the vena cava and ascending aorta. Upon opening the RA, a large mass originating from the Eustachian valve was identified and successfully resected (Figure 4).

A full-thickness section of the RA, where the mass had originated, was also excised to ensure complete removal. The incision site was closed with a pledgetted 4-0 Prolene suture since the RA was dilated and redundant. The patient tolerated the operation without the need for any blood products and was weaned from CPB with no complications. She was extubated successfully in the cardiac intensive care unit after four hours and remained hemodynamically stable during her stay. The drains and wires were removed on post-operative day (POD) 2. She had no postoperative issues and progressed as expected to the cardiac step-down unit, where she was subsequently discharged from the hospital on POD 4. She followed up in the clinic at one week and one month postoperatively with no reported complications or issues, noting that she was doing well with an improvement in her shortness of breath and cough.

## Discussion

Previous studies have discussed details on anesthetic management for cardiac myxoma patients, but have not extensively explored initiating CPB before the induction of general anesthesia. In managing cardiac myxomas, the primary goal of intraoperative anesthesia is to closely monitor and maintain the systemic blood pressure in a normotensive range through the use of IV fluids and vasoactive medications [3]. Some cases have reported the use of crystalloids to maintain preload while avoiding inotropes to prevent further obstruction of the tricuspid valve [4], whereas others employed intravenous infusions of dopamine or milrinone to maintain hemodynamic stability [5].

In cases involving a mass obstructing the tricuspid valve, general anesthesia may precipitate cardiovascular collapse upon induction. A 2023 study examining 110 patients with cardiac myxomas found that 48 patients (43.6%) had tumors prolapsing into the ventricle, obstructing the tricuspid or mitral valve [5]. Among these, 38 patients (34.5%) experienced hemodynamic instability during the induction of general anesthesia [5]. With over a third of patients acutely decompensating, the use of CPB before anesthesia induction emerges as a potential strategy in such high-risk cases.

A review of the literature reveals limited studies on preoperative CPB initiation. Notable examples include a case involving an anterior mediastinal mass [6] and another concerning a ruptured ascending aortic aneurysm [2]. In the case of the mediastinal mass, severe airway and cardiovascular compression precluded safe induction of general anesthesia. As a result, the team initiated femoral CPB at 0.5 L/min under local lidocaine. This was followed by inhalation induction with sevoflurane. Despite this precaution, blood pressure dropped immediately after induction, necessitating full activation of CPB for stabilization. This approach highlights how initiating CPB prior to general anesthesia can help maintain hemodynamic control and reduce the risk of acute decompensation in critical scenarios.

For clinicians considering the use of CBP before general anesthesia, several important factors should be considered. First, local anesthetic nerve blocks carry the risk of patient agitation. In our case, a local anesthetic was utilized at the access points, but a full nerve block was not required. This provided adequate analgesia, and further interventions were not necessary. Second, there are risks regarding inferior vena cava (IVC) cannulation. Our patient was placed on CPB via a multi-stage IVC cannula prior to the induction of anesthesia. After induction and sternotomy, a superior vena cava cannula was placed, and the patient was converted to bi-caval cannulation. When placing the IVC cannula, great care was taken, and an echocardiogram was utilized to ensure the cannula did not enter the RA and disrupt the mass. Third, there are alternative approaches to CPB and general anesthesia. While we agree that venoarterial extracorporeal membrane oxygenation (VA-ECMO) has its advantages, this patient would ultimately require full CPB for mass resection, and VA-ECMO converted to full CPB after sternotomy risks complications.

Initiating CPB before general anesthesia is not without drawbacks. One major consideration is the use of systemic heparin to enable femoral catheterization [2]. Administering systemic heparin before a sternotomy increases bleeding risk during surgery, potentially resulting in greater blood loss and an elevated need for transfusion. Without the stabilizing effects of anesthesia, initiating CPB can lead to hemodynamic instability. Hypotension and cardiovascular collapse are complications that can occur due to sudden changes in preload and afterload [7]. Pulmonary complications such as hypoxemia and acute respiratory distress syndrome may arise due to inadequate control of ventilation and oxygenation [8]. Airway edema may occur from priming fluids or prolonged time on the pump, which can complicate intubation and extubation. Additionally, cannulation complications may arise due to patient movement and lack of muscle relaxation that would be resolved with general anesthesia. These complications may include malposition of cannulas, dissection, and gas embolism [9]. Lastly, it is important to consider patient comfort, as initiation of CPB before general anesthesia can cause anxiety and distress [9]. While these trade-offs are significant, the hemodynamic stability achieved through preoperative CPB can be crucial in cases where patients are at high risk of collapse during surgery. 

## Conclusions

Ultimately, preoperative CPB is a valuable tool that should be considered in select cases that pose a high risk of hemodynamic instability, particularly when conventional anesthesia management poses substantial risks. Balancing the benefits of improved hemodynamic stability against the risks of increased bleeding and other sequelae is essential when deciding to implement this approach.
